# Body mass index, lung function, and FeNO in children with positive bronchodilator response: a cross-sectional study

**DOI:** 10.3389/fped.2026.1849715

**Published:** 2026-07-31

**Authors:** Tingting Zhou, Juan Liu, Yuanyuan Lin, Guilan Zhang, Yaqi Chen, Xiao Liu, Jun Guo, Xiaofang Wu

**Affiliations:** Department of Pediatrics, Mianyang Central Hospital, Mianyang, Sichuan, China

**Keywords:** body mass index, children, lung function, obesity, pulmonary function test

## Abstract

**Objective:**

To investigate the association between body mass index (BMI) and pulmonary function in pediatric outpatients with positive bronchodilator response. We hypothesized that BMI is associated with impaired pulmonary function in this population.

**Methods:**

This cross-sectional study was conducted at the Department of Pediatrics of Mianyang Central Hospital from January 2024 to December 2024. A total of 430 children aged 4–14 years who met the inclusion criteria were enrolled. Based on body mass index (BMI), participants were categorized into an higher BMI group (*n* = 78), a normal BMI group (*n* = 338), and an lower BMI group (*n* = 14). Pulmonary function tests were performed to measure forced vital capacity (FVC), forced expiratory volume in 1 s (FEV_1_), FEV_1_/FVC ratio, maximum mid-expiratory flow (MMEF), and forced expiratory flow at 25%, 50%, and 75% of FVC (FEF_25_, FEF_50_, FEF_75_). Bronchodilator responsiveness was evaluated after salbutamol inhalation by measuring changes in FEV_1_ and FVC, including absolute and percentage increases. Fractional exhaled nitric oxide (FeNO) levels were measured using standardized procedures. Correlations between BMI, FeNO levels, and pulmonary function parameters were analyzed. Statistical analysis was performed using appropriate tests. A two-tailed *P* value < 0.05 was considered statistically significant.

**Results:**

No significant differences were observed in baseline pulmonary function parameters, including FVC% predicted, FEV_1_% predicted, FEV_1_/FVC, MMEF, FEF_25_, FEF_50_, and FEF_75_, across BMI groups (all *P* > 0.05). A significant difference in FEV_1_/FVC was observed between boys and girls (*P* < 0.05). After bronchodilator testing, a significant difference was observed only in the absolute increase in FVC among BMI groups (*P* = 0.016), whereas no significant difference was found in FEV_1_ improvement. Fractional exhaled nitric oxide (FeNO) levels were positively correlated with the absolute increase in FVC (*r* = 0.165, *P* = 0.002).

**Conclusion:**

No significant differences were observed in baseline pulmonary function parameters across BMI groups in children aged 4–14 years. However, differences in bronchodilator-induced changes in FVC were identified among different BMI categories, and FeNO levels were positively associated with bronchodilator-induced changes in FVC. These findings indicate that body mass index and FeNO are independently associated with bronchodilator-related changes in lung function in this population, which may be relevant when interpreting pediatric pulmonary function test results.

## 1 Introduction

In recent years, the incidence of respiratory diseases in children has increased, becoming one of the major public health concern. Pulmonary function testing, as a commonly used tool for assessing respiratory status in children, plays a key role in the early identification of abnormal respiratory function (1). Meanwhile, body mass index (BMI), a widely used indicator reflecting physical growth status in children, has increasingly been shown to be closely associated with respiratory health (2).

An increasing number of studies have focused on the impact of obesity on lung function. Previous studies have suggested that overweight or obesity may influence pulmonary function through inflammatory mechanisms, alterations in respiratory mechanics, and other pathways (3).

However, most existing studies primarily focus on the relationship between childhood asthma and lung function, with relatively few studies including children with bronchodilator responsiveness as the study population. In addition, previous research has largely concentrated on the effects of overweight or obesity on lung function, with limited attention paid to children with low BMI (1–6). Pulmonary function parameters, including FEV_1_, FVC, MMEF, and FEF values, are commonly used to assess airway obstruction and ventilatory function in children. Bronchodilator responsiveness reflects the reversibility of airway limitation and is widely used in the assessment of pediatric respiratory diseases. Fractional exhaled nitric oxide (FeNO), a noninvasive biomarker associated with airway inflammation, has been reported to correlate with bronchodilator responsiveness and pulmonary function changes in children with respiratory diseases (7). Therefore, there remains a need to further investigate the association between BMI and pulmonary function, as well as the role of FeNO in pulmonary function changes in outpatients.

Based on previous evidence, we hypothesized that BMI statuses are associated with differences in pulmonary function responsiveness in children with positive bronchodilator response. Therefore, the primary aim of this study was to investigate the relationship between pulmonary function parameters and BMI among pediatric outpatients who underwent pulmonary function testing at Mianyang Central Hospital in 2024. A secondary objective was to explore the relationship between fractional exhaled nitric oxide (FeNO) levels and pulmonary function parameters. The findings of this study may help improve the interpretation of pulmonary function test results in children with different BMI statuses and provide additional evidence regarding the relationship between nutritional status and respiratory function in pediatric populations. The results may also be of interest to public health practitioners and researchers focusing on child growth and respiratory development.

## 2 Subjects and methods

### 2.1 Study population

A total of 430 children aged 4–14 years who attended the pediatric outpatient department of Mianyang Central Hospital between January and December 2024 and demonstrated a positive bronchodilator response on pulmonary function testing were included in this retrospective study. Pulmonary function tests were performed in the pulmonary function laboratory of Mianyang Central Hospital under standardized indoor environmental conditions according to routine institutional procedures. Among them, 267 were boys and 163 were girls. The age of the participants ranged from 4 to 14 years, with a mean age of 7.15 ± 2.25 years. The inclusion criteria were as follows: (1) children who were able to complete standardized pulmonary function tests with acceptable test quality; (2) absence of severe systemic diseases (such as cardiovascular or neurological disorders) during initial or follow-up visits that could interfere with pulmonary function measurements.

The exclusion criteria were as follows: (1) presence of congenital cardiopulmonary diseases, neuromuscular disorders, or other major underlying diseases that might affect pulmonary function assessment; (2) acute respiratory tract infection on the day of testing, or symptoms such as fever or severe cough; (3) missing key variables or incomplete clinical data.

### 2.2 Methods

Data collected included sex, age, height, weight, history of allergy (including food allergy history and positive allergen sensitization results), pulmonary function parameters, and bronchodilator test results. Given the relatively large sample size, parametric statistical methods were applied.

#### 2.2.1 Pulmonary function testing

All participants underwent pulmonary function testing using the same spirometer (Jaeger MasterScreen, Germany), operated in accordance with standardized procedures and relevant guidelines. Before testing, children were instructed to relax and adjust their breathing. After achieving stable breathing, pulmonary function testing was performed. Each test was repeated three times, and the best result was selected and recorded. The pulmonary function parameters measured in this study included forced vital capacity (FVC), forced expiratory volume in 1 s (FEV_1_), the measured FEV_1_/FVC ratio, maximum mid-expiratory flow (MMEF), and forced expiratory flow at 25%, 50%, and 75% of vital capacity (forced expiratory flow, FEF_25_, FEF_50_, and FEF_75_). Measured values were expressed as percentages of predicted values (% predicted). After completion of routine pulmonary function testing, a bronchodilator test was performed using nebulized 0.20% salbutamol sulfate solution. The dose was adjusted according to age: 1.25 mL for children aged <6 years, 1.875 mL for those aged 6–12 years, and 2.5 mL for those aged >12 years. Changes in measured FVC and FEV_1_, as well as the percentage increases in predicted FVC% and FEV_1_%, were recorded to assess airway reversibility. Fractional exhaled nitric oxide (FeNO) was measured using a standardized online analyzer under tidal breathing conditions. FeNO levels were expressed in parts per billion (ppb) and used as a noninvasive marker of airway inflammation.

#### 2.2.2 Body mass index

Height and weight were measured by trained respiratory specialists using standardized measuring instruments by Changchun Jinsai Pharmaceutical Co., Ltd. (Changchun, China). Children were measured without shoes and in light clothing. Height was measured in a standing position using a stadiometer, and weight was measured using a digital weighing scale. Each measurement was taken three times, and the average value was recorded. BMI was calculated as weight (kg) divided by height squared (m^2^). BMI was standardized within age- and sex-specific groups using internal percentile distributions. Participants were categorized based on the distribution of BMI percentiles, with cut-offs defined at the 5th and 85th percentiles to identify relatively low, normal, and high BMI groups within the study population (8, 9).

### 2.3 Statistical analysis

Statistical analysis was performed using STATA MP version 17.0. Categorical variables were expressed as frequencies and percentages, and were analyzed using the chi-square test. Continuous variables were described as mean ± standard deviation. Comparisons of means among multiple groups were conducted using one-way analysis of variance (ANOVA), and pairwise comparisons between two groups were performed using the least significant difference (LSD) test. Pearson correlation analysis was applied to assess the correlations between pulmonary function parameters and BMI. A *P* value < 0.05 was considered statistically significant.

## 3 Results

### 3.1 Baseline characteristics

A total of 430 children were included in the analysis, comprising 267 boys (62%) and 163 girls (38%). According to BMI classification, 14 children (3.26%) were assigned to the lower BMI group, 338 (78.79%) to the Normal BMI group, and 78 (18.18%) to the Higher BMI group. The age of the participants ranged from 4 to 14 years, with a mean age of 7.14 ± 2.25 years. No significant differences were observed among the three BMI groups in sex distribution, age, or allergy history (*P* > 0.05) (Table 1).

**Table 1:** Comparison of baseline characteristics among pediatric outpatients with different BMI categories.

Group	Lower BMI groups (*n* = 14)	Normal BMI groups (*n* = 338)	Higher BMI groups (*n* = 78)	*χ*^2^/*F*	*P*
Sex (%)				1.69	0.429
Boys	11 (78.57)	208 (61.54)	48 (61.54)		
Girls	3 (21.43)	130 (38.46)	30 (38.46)		
Age	6.64 ± 1.69	7.10 ± 2.22	7.42 ± 2.46	1.00	0.368
Allergy history [*n* (%)]	5 (35.71)	40 (15.21)	7 (10.61)	1.37	0.059

### 3.2 Comparison of pulmonary function parameters among pediatric outpatients

No statistically significant differences were observed in FVC % predicted, FEV_1_% predicted, FEV_1_/FVC (%), MMEF, FEF_25_, FEF_50_, or FEF_75_ among the three BMI groups (all *P* > 0.05) (Table 2). As shown in Table 3, there were no statistically significant differences in FVC % predicted, FEV_1_% predicted, MMEF, FEF_25_, FEF_50_, or FEF_75_ between boys and girls (all *P* > 0.05). In contrast, FEV_1_/FVC (%) was significantly higher in girls than in boys.

**Table 2:** Pulmonary function parameters by BMI in pediatric outpatients.

Item	Lower BMI groups	Normal BMI groups	Higher BMI groups	*F*	*P*
FVC % predicted	89.56 ± 14.07	92.34 ± 12.32	92.16 ± 14.52	0.32	0.728
FEV_1_% predicted	80.82 ± 15.24	82.73 ± 11.74	82.02 ± 12.64	0.26	0.771
FEV_1_/FVC (%)	89.17 ± 6.98	89.93 ± 6.68	89.01 ± 6.40	0.65	0.523
MMEF	48.20 ± 13.04	50.23 ± 10.02	49.77 ± 9.45	0.32	0.724
FEF_25_	41.15 ± 11.64	42.62 ± 9.69	42.01 ± 9.53	0.26	0.772
FEF_50_	49.59 ± 13.41	51.45 ± 10.36	51.03 ± 9.73	0.25	0.778
FEF_75_	63.64 ± 15.70	65.70 ± 12.37	64.74 ± 12.51	0.34	0.712

**Table 3:** Correlation between sex and pulmonary function parameters in pediatric outpatients.

Item	Boys	Girls	*F*	*P*
FVC % predicted	91.71 ± 12.73	93.01 ± 12.89	1.06	0.304
FEV_1_% predicted	82.12 ± 12.67	83.22 ± 10.87	0.84	0.359
FEV_1_/FVC (%)	75.85 ± 5.70	77.85 ± 5.57	12.66*	<0.001
MMEF	50.12 ± 9.68	50.03 ± 10.58	0.01	0.924
FEF_25_	42.3 ± 9.11	42.74 ± 10.69	0.20	0.653
FEF_50_	51.22 ± 10.07	51.47 ± 10.83	0.06	0.813
FEF_75_	66.14 ± 13.05	66.36 ± 11.51	2.07	0.151

* Statistically significant difference among groups (*P* < 0.05).

### 3.3 Comparison of bronchodilator test results among the three BMI groups

**Figure 1:**
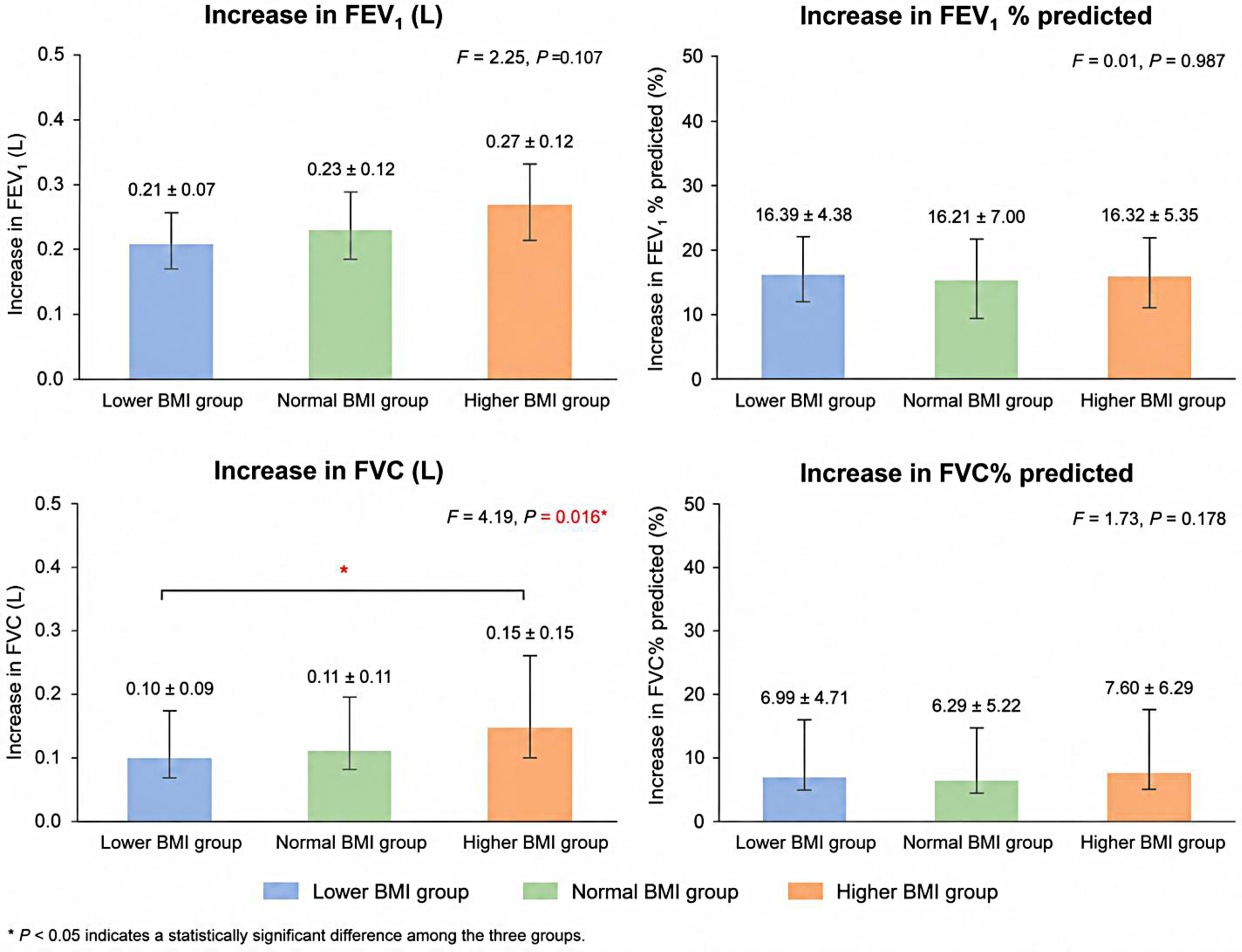
Comparison of bronchodilator test results in pediatric outpatients.

Twenty minutes after inhalation of a bronchodilator, differences in post-intervention improvement in pulmonary function were observed across BMI groups (Figure 1). A statistically significant difference was found in the absolute increase in FVC among the three BMI groups (*F* = 4.19, *P* = 0.016), with the Higher BMI group showing a greater increase compared with the other groups. No significant differences were observed in the increases in FEV_1_, FEV_1_% predicted, or FVC% predicted among BMI groups (all *P* > 0.05; Figure 1). Overall, bronchodilator administration resulted in improvement in pulmonary function across all BMI categories, while BMI status was associated only with absolute FVC response.

### 3.4 Correlation between fractional exhaled nitric oxide (FeNO) and post-bronchodilator lung function improvement

Correlation analyses were conducted to assess the association between FeNO levels and bronchodilator responsiveness (Table 4). FeNO levels were positively correlated with the absolute increases in FEV_1_ (*r* = 0.234, *P* < 0.001) and FVC (*r* = 0.165, *P* = 0.002) after bronchodilator inhalation. However, no significant correlations were observed between FeNO levels and improvements expressed as predicted percentage values, including changes in FEV_1_% predicted and FVC% predicted (both *P* > 0.05).

**Table 4:** Correlation between fractional exhaled nitric oxide (FeNO) and post-bronchodilator lung function improvement.

Lung function improvement indices	Correlation coefficient (*r*)	*P*
Increase in FEV_1_	0.234*	<0.001
Increase in FEV_1_ % predicted	0.055	0.313
Increase in FVC	0.165*	0.002
Increase in FVC% predicted	0.068	0.211

* Statistically significant difference among groups (*P* < 0.05).

## 4 Discussion

Globally, the burden of pediatric asthma and wheezing disorders remains substantial, while issues of undernutrition and overweight are increasingly intertwined, creating a complex health landscape. Obesity-related asthma was proposed in 2014, and previous studies have indicated that obesity is a risk factor for asthma, accounting for 23%–27% of pediatric asthma cases (2). The impact of obesity on asthma has been widely studied.

Unlike many previous studies that primarily focused on obesity or asthma populations, in this hospital-based pediatric outpatient population with positive bronchodilator response, we found that baseline spirometric parameters did not differ significantly across BMI categories. However, BMI was associated with differences in bronchodilator-induced changes in FVC, while no significant differences were observed for FEV_1_ or percentage predicted indices. In addition, FeNO levels were positively associated with absolute improvements in FEV_1_ and FVC following bronchodilator administration.

The relationship between pulmonary function parameters and BMI remains controversial. Some studies suggest that in overweight and obese individuals, excessive fat accumulation in the abdominal cavity, diaphragm, and chest wall may lead to diaphragmatic elevation, restrict chest wall mobility, and reduce lung compliance, thereby increasing airway resistance (10) and decreasing lung volumes, FEV_1_, FVC, and other parameters (11). However, other studies report no significant association between overweight or obesity and lung function in children (12). On the other hand, some studies have found that underweight is also associated with reduced lung function, which may be related to insufficient pulmonary development. Low body weight during childhood (*P* < 0.001) has been linked to restricted lung function in adulthood (13, 14). In addition, BMI below the 10th percentile at 11 years of age can predict restricted lung function in adulthood (RRR = 3.66; 95% CI: 1.48–9.02; *P* = 0.0048) (13). No significant associations were observed between BMI and these parameters in our study. Previous guidelines have indicated that these mid-expiratory flow indices are highly variable and influenced by lung volume and effort, particularly in pediatric populations, which may limit their interpretability in epidemiological studies (3, 15). Although MMEF and FEF_25%–75%_ are considered sensitive to early changes in small airway function, their interpretation should be made with caution due to their high variability, effort dependence, and limited reproducibility, particularly in pediatric populations (3).

In addition, FEV_1_/FVC was significantly higher in girls than in boys, which is consistent with previous studies. A higher FEV_1_/FVC ratio in girls suggests a faster lung emptying rate and a shorter expiratory time constant, supporting the concept that airway development occurs earlier in girls than in boys (16, 17). These sex-related differences indicate that sex may act as a potential modifying factor when interpreting the relationship between BMI and pulmonary function in children. Accordingly, sex-specific considerations may be warranted in pediatric pulmonary health management to improve the precision of clinical guidance.

More importantly, this study further compared bronchodilator responsiveness among pediatric children with different BMI categories. The results showed that a significant difference was observed in the increase in absolute FVC values after bronchodilator administration, while no statistically significant difference was found in FEV_1_ improvement among BMI groups. These findings suggest that Low BMI children exhibited relatively smaller improvements in key pulmonary function parameters, such as FVC. Previous studies have reported that pulmonary function indicators, including FVC, FEV_1_, FVC% predicted, and inspiratory capacity, are generally lower in underweight individuals than in those with normal weight, overweight, or obesity. In addition, underweight patients with bronchiectasis tend to have more severe disease (18). The underlying mechanisms may be related to nutritional status and growth and development. Low body weight may reflect insufficient nutritional reserves and impaired somatic development, which could adversely affect respiratory muscle strength and pulmonary growth. And long-term insufficient energy and protein intake may reduce respiratory muscle mass and strength, thereby limiting improvements in lung volumes and expiratory flow rate (19). In addition, malnutrition has been associated with impaired immune function, which may increase the risk of recurrent respiratory infections, contribute to pulmonary function impairment, and elevate the risk of developing asthma in adulthood, while also adversely affecting physical and psychological health (20). In contrast, children with normal weight or mild overweight generally have more adequate nutritional and hormonal status, and their thoracic and pulmonary development is closer to the normal trajectory, which may partly contribute to differences in bronchodilator responsiveness across BMI categories (21).

This study also examined the association between airway inflammation and bronchodilator responsiveness. The results showed that children with higher fractional exhaled nitric oxide (FeNO) levels exhibited greater absolute increases in FEV_1_ and FVC following bronchodilator administration, suggesting that children with more active airway inflammation may have more pronounced reversible airflow limitation in this hospital-based pediatric outpatient population. Eosinophilic airway inflammation may be associated with increased airway hyperresponsiveness and reversibility, which could partly explain the greater bronchodilator responsiveness observed in children with elevated FeNO levels. This finding may be related to characteristics of type 2 (T2)-high inflammation, which is commonly represented by eosinophilic airway inflammation. Therefore, FeNO may serve as a potential biomarker for predicting bronchodilator responsiveness in children. However, the lack of a significant association between FeNO levels and relative improvements expressed as predicted percentage values indicates that both absolute and relative indices may provide complementary information when interpreting bronchodilator response in this population.

Overall, our findings suggest that BMI status is more closely related to dynamic pulmonary responsiveness than to baseline lung function in children with positive bronchodilator response. Nutritional status and airway inflammation may therefore jointly influence variability in bronchodilator-induced changes in lung function.

These findings have several practical implications for clinical practice and pediatric respiratory health management. In children undergoing bronchodilator testing, interpretation of spirometric reversibility should take into account body mass index, as BMI status may influence the magnitude of absolute lung function changes, particularly FVC responses. This is especially relevant when evaluating children with lower BMI, in whom smaller absolute improvements may reflect differences in growth and respiratory muscle development rather than reduced airway responsiveness. In addition, fractional exhaled nitric oxide (FeNO) may provide useful complementary information regarding airway inflammatory status and help contextualize bronchodilator responsiveness in clinical interpretation. Sex-related differences in FEV_1_/FVC further suggest that sex should be considered as a physiological factor when interpreting baseline spirometric indices in children. From a broader health management perspective, these findings highlight the importance of integrating nutritional status and airway inflammation assessment into routine pediatric respiratory evaluation. This may improve the precision of lung function interpretation in outpatient settings and support more individualized assessment of pediatric respiratory health.

This study has several strengths. First, unlike many previous studies focusing primarily on obesity or asthma populations, this study additionally included low BMI children and evaluated a hospital-based pediatric outpatients with positive bronchodilator response. Second, multiple respiratory-related parameters, including pulmonary function, bronchodilator responsiveness, and FeNO levels, were comprehensively assessed. These findings may provide additional evidence for understanding the association between BMI status and pulmonary function responsiveness in children.

## 5 Limitations

This study has several limitations. First, body composition and fat distribution were not assessed due to lack of data. Second, metabolic and dietary factors were not available, which may have influenced the observed associations. Third, potential confounding factors such as environmental exposures could not be fully controlled due to the retrospective design. In addition, the BMI classification approach was based on internal percentile distributions and may limit direct comparison with studies using WHO or CDC reference standards. Finally, the cross-sectional nature of the study precludes causal inference. Future longitudinal studies with larger sample sizes are needed to further clarify these associations.

## Data Availability

The raw data supporting the conclusions of this article will be made available by the authors upon reasonable request.
